# Analysis of prognostic prediction and nursing intervention value in PD patients based on nomogram model

**DOI:** 10.3389/fmed.2025.1645631

**Published:** 2025-12-15

**Authors:** Yugang Cao, Tao Fang, Jun Guo, Xun Hu, Jin Yang

**Affiliations:** Hubei Key Laboratory for Kidney Disease Pathogenesis and Intervention, Department of Hepatobiliary Surgery, Huangshi Central Hospital, Hubei Polytechnic University School of Medicine, Huangshi, Hubei, China

**Keywords:** peritoneal dialysis, technical survival, nomogram, prognostic prediction, nursing intervention

## Abstract

**Objective:**

To explore the independent risk factors for technical survival in patients undergoing peritoneal dialysis (PD), construct a nomogram model for predicting 1-, 3-, and 5-year technical survival rates, and validate the clinical value of nursing interventions in improving PD patients’ prognosis.

**Methods:**

A retrospective cohort study was conducted on 475 patients with end-stage renal disease (ESRD) who received PD at Huangshi Central Hospital from February 2010 to April 2025. Patients were randomly divided into a training group (*n* = 332) and a validation group (*n* = 143) at a 7:3 ratio. Spearman correlation analysis was used to identify factors associated with PD duration. Univariate and multivariate Cox regression analyses were performed to screen independent risk factors for PD technical survival, based on which a nomogram prediction model was established. The model’s performance was validated using receiver operating characteristic (ROC) curves, concordance index (C-index), calibration plots, and decision curve analysis (DCA). Kaplan–Meier method was applied to analyze cumulative risk differences related to nursing intervention factors.

**Results:**

Spearman correlation analysis showed that PD duration was negatively correlated with age, body mass index (BMI), fasting blood glucose, serum creatinine (Scr), peritonitis, catheter-related complications, Self-Rating Depression Scale (SDS) score, and Self-Rating Anxiety Scale (SAS) score, while positively correlated with years of education, DUV, albumin level, number of primary caregivers, and frequency of health education (all *p* < 0.05). Multivariate Cox regression analysis identified six independent predictors of PD technical survival: age ≥60 years (hazard ratio [HR] = 9.084, 95% confidence interval [CI]:5.912–13.959), history of diabetes mellitus (HR = 15.047, 95%CI:9.802–23.101), albumin level (HR = 0.894, 95%CI:0.849–0.940), peritonitis (HR = 6.172, 95%CI:3.970–9.595), catheter-related complications (HR = 1.740, 95%CI:1.304–2.320), and abnormal mental state (HR = 2.261, 95%CI:1.589–3.217) (all *p* < 0.01). The nomogram constructed based on these factors showed good predictive performance in both the training and validation groups.

**Conclusion:**

The constructed nomogram can accurately predict the 1-, 3-, and 5-year technical survival rates of PD patients. Enhanced health education (≥1 session/month), optimized caregiving support systems, improved psychological conditions, and narrowed urban–rural disparities in healthcare resources are effective nursing interventions to improve the technical survival outcomes of PD patients.

## 1 Introduction

PD is widely utilized globally as a crucial replacement therapy for ESRD (1). However, technical survival among PD patients is influenced by multiple complex factors, including underlying diseases, complications, psychological conditions, and nursing interventions, significantly increasing uncertainty in prognosis evaluation and clinical decision-making. Herein, technical survival refers to the proportion of PD patients who can continue PD treatment within a specific period (e.g., 1, 3, or 5 years) without being forced to switch to hemodialysis or discontinue dialysis due to technique-related failures—such as recurrent peritonitis, catheter dysfunction (e.g., obstruction or infection), or peritoneal function decline—which is distinct from overall patient survival that focuses on mortality outcomes (2–4).

Current studies on PD prognostic models have notable limitations: most single-variable prognostic models lack external validation, affecting their reliability in broader clinical settings; some nomogram-based prediction models, despite being applied in practice, fail to incorporate psychological factors—despite sufficient evidence confirming their significant impact on treatment adherence. Additionally, relevant systematic reviews indicate that only a small proportion of existing models integrate social support-related variables, revealing obvious gaps in the coverage of multidimensional risk factors.

This study adopted Donabed’s structure-process-outcome model to guide variable selection: structural variables include demographic (age, residence) and clinical characteristics (diabetes history, albumin level); process variables encompass nursing interventions (health education frequency, caregiver support); and outcome variables focus on technical survival and related clinical outcomes.

Existing evidence indicates that nursing interventions such as health education, psychological support, and family accompaniment may improve clinical outcomes in PD patients, but these terms lack standardization. All interventions in this study were developed based on the 2022 International Society for Peritoneal Dialysis (ISPD) Guidelines (5), with key components (e.g., health education content: PD operation standardization, complication prevention; psychological support: cognitive-behavioral therapy; caregiver training: daily care skills), duration (each session 30–45 min), and frequency detailed in Supplementary material 1.

To address current research gaps, we proposed two testable hypotheses: (1) The nomogram constructed by integrating age, diabetes history, albumin level, peritonitis, catheter-related complications, and mental state will accurately predict 1-, 3-, and 5-year technical survival rates of PD patients; and (2) Structured nursing interventions (≥1 health education session/month, psychological support for abnormal mental state, and optimized caregiver support) will significantly reduce the risk of technical failure (primary outcome) and improve secondary outcomes including hospitalization frequency and SF-36 quality of life scores. Therefore, this study aims to identify independent risk factors influencing technique survival among PD patients, construct and validate a nomogram-based risk prediction model, and systematically evaluate the clinical value of standardized nursing interventions, thereby providing scientific evidence and practical guidance for optimizing the management system of PD patients.

## 2 Materials and methods

### 2.1 Study subjects

We retrospectively selected the patient cohort for this study. All data were collected from patients with end-stage renal disease who received PD at Huangshi Central Hospital between February 2010 and April 2025. This study was approved by the Institutional Ethics Committee of Huangshi Central Hospital (Approval No.: Lun Kuai Shen [2025]-48), and all patient identifiers were anonymized prior to analysis; therefore, the requirement for informed consent was waived.

The research team accessed medical records of all patients with end-stage renal disease who underwent PD at Huangshi Central Hospital during the study period. Inclusion criteria were as follows: (1) age between 14 and 75 years; (2) no history of malignancy; (3) diagnosis of end-stage renal disease; and (4) absence of severe cognitive impairment. Exclusion criteria were as follows: (1) incomplete baseline data; (2) presence of malignancy or other comorbidities with an expected survival time <6 months; (3) participation in other interventional clinical trials within the past 3 months; and (4) pregnancy or lactation. A total of 475 eligible patients were enrolled in the study and randomly assigned to either the training group or validation group at a ratio of 7:3, resulting in 332 patients in the training group and 143 patients in the validation group.

### 2.2 Data collection

We retrospectively collected medical records for all peritoneal dialysis patients with end-stage renal disease through the electronic medical record HIS system. Collected variables included:

Demographic and clinical characteristics: Age, gender, BMI, marital status, years of education (defined as total years of formal schooling received, e.g., 9 years for junior high school graduation, 12 years for senior high school graduation), type of health insurance, residence (urban/rural, based on the patient’s registered household address and long-term living place), peritoneal dialysis mode (PDM: automated peritoneal dialysis [APD] vs. continuous ambulatory peritoneal dialysis [CAPD]), history of diabetes (defined as a prior clinical diagnosis of type 1 or type 2 diabetes mellitus, or fasting blood glucose ≥7.0 mmol/L on two consecutive measurements), number of episodes of catheter-related complications (e.g., catheter obstruction, exit-site infection, peritoneal effluent leakage), number of episodes of peritonitis during dialysis (diagnosed according to the 2022 ISPD Guidelines: presence of at least two of the following: abdominal pain, cloudy peritoneal effluent, effluent white blood cell count >100/μL with >50% neutrophils), serum creatinine (Scr, average before initiation of peritoneal dialysis), daily ultrafiltration volume (DUV, average during peritoneal dialysis), and albumin levels (average during peritoneal dialysis, measured by bromocresol green method).
Mental status assessment: Evaluated using the SDS and SAS, which are validated psychological assessment tools widely used in clinical practice.
SDS: A 20-item scale with total scores ranging from 20 to 80; scores are converted to standard scores (standard score = raw score × 1.25). A standard score <53 indicates normal depression status, while ≥53 indicates abnormal depression status (mild: 53–62, moderate: 63–72, severe: >72).
SAS: A 20-item scale with total scores ranging from 20 to 80; scores are converted to standard scores using the same formula. A standard score <50 indicates normal anxiety status, while ≥50 indicates abnormal anxiety status (mild: 50–59, moderate: 60–69, severe: >69).

For this study, abnormal mental state was defined as meeting either “SDS standard score ≥53” or “SAS standard score ≥50”; normal mental state required both “SDS standard score <53” and “SAS standard score <50.”

Health education-related variables:

Health education content: Standardized according to the 2022 ISPD Guidelines, including 4 core modules: PD operation standardization (e.g., aseptic technique for dialysate exchange, catheter care), complication prevention (recognition and first-aid of peritonitis, management of fluid overload), dietary guidance (low-salt, low-potassium, high-quality protein diet), and medication adherence (usage and precautions of erythropoietin, phosphate binders). Each education session lasted 30–45 min, delivered by trained nephrology nurses via face-to-face lectures or video tutorials.
Frequency of health education: Defined as the number of standardized health education sessions received per month. For statistical analysis, patients were divided into two groups: ≥1 time/month (regular health education, including monthly routine sessions or additional sessions based on individual needs, e.g., patients with poor operation compliance) and <1 time/month (irregular or no health education, e.g., patients who missed sessions due to long-distance residence or poor follow-up adherence).

### 2.3 Follow-up

Follow-up was conducted using a combination of regular telephone calls and outpatient visits. Trained researchers performed monthly telephone interviews, collecting systematically detailed clinical information regarding patients’ daily living conditions, dietary management, medication adherence, and presence of any adverse symptoms, which were recorded in standardized follow-up forms. Outpatient follow-up was scheduled according to individual patient conditions, with a frequency tailored to each case (every 3 months or 6 months). Patients are required to visit Huangshi Central Hospital for comprehensive evaluation, including biochemical tests such as serum creatinine, albumin, and DUV, as well as monitoring of PD-related complications, such as the frequency of peritonitis episodes and catheter-related complications. Additionally, psychological status assessments using the Self-Rating Depression Scale (SDS) and Self-Rating Anxiety Scale (SAS) are conducted every 6 months to 1 year. Based on the scores, appropriate psychological interventions are provided, with referrals to psychiatry for further evaluation and treatment when necessary.

### 2.4 Statistical analysis

Continuous variables were tested for normality using the Shapiro–Wilk test; those not following a normal distribution (e.g., DUV, Scr, albumin, SDS score) are expressed as median (interquartile range) [M (Q1–Q3)], and normally distributed variables (if any) as mean ± standard deviation (±SD). Categorical variables are reported as frequencies (percentages). Spearman correlation analysis was used to explore associations between peritoneal dialysis vintage (non-normal distribution) and clinical variables. Univariate Cox regression was performed to screen potential variables (*p* < 0.1), followed by multivariate Cox proportional hazards modeling to identify independent risk factors, calculating hazard ratios (HR) and 95% confidence intervals. A nomogram predicting 1-, 3-, and 5-year technique survival rates was developed based on these independent risk factors. Model validation included assessment of discrimination using the area under the receiver operating characteristic curve (AUC) and concordance index (C-index), calibration curves to evaluate agreement between predicted and observed outcomes, and decision curve analysis (DCA) to quantify clinical net benefit. Risk stratification based on total nomogram scores was performed using the maxstat test method, Kaplan–Meier survival curves were used to compare differences between groups, and Log-rank tests analyzed cumulative risks associated with nursing intervention factors. Statistical analyses were performed using SPSS 26.0 (univariate and multivariate Cox regression) and R 4.2.1 (modeling and visualization). A two-sided *p*-value <0.05 was considered statistically significant.

## 3 Results

### 3.1 Baseline characteristics

According to the inclusion and exclusion criteria, a total of 475 patients with end-stage renal disease treated by PD were included in this study. Among them, 332 cases were in the training cohort and 143 cases were in the validation cohort. All patients were treated with PD. Follow-up data showed that there were 197 males and 135 females in the training cohort, 84 males and 59 females in the verification cohort, 203 training cohorts and 98 verification cohorts aged ≥ 60, 129 training cohorts and 45 verification cohorts aged <60. Table 1 presents the demographic and clinical characteristics of patients on PD with end-stage renal disease in the training and validation cohort.

**Table 1:** Baseline characteristics of end-stage renal disease patients undergoing PD in the training and validation cohort.

Variable		Training cohort (*n* = 332)	Validation cohort (*n* = 143)
Gender	Male	197 (59.3%)	84 (58.7%)
	Female	135 (40.7%)	59 (41.3%)
Age	≥60	203 (61.1%)	98 (68.5%)
	<60	129 (38.9%)	45 (31.5%)
BMI	<18.5	48 (14.5%)	21 (14.7%)
	18.5–22.9	57 (17.2%)	30 (21%)
	23–24.9	31 (9.3%)	10 (7%)
	≥25	196 (59%)	82 (57.3%)
Marital status	Yes	263 (79.2%)	117 (81.8%)
	No	69 (20.8%)	26 (18.2%)
Education years (years)	≤9	198 (59.6%)	84 (58.7%)
	>9	134 (40.4%)	59 (41.3%)
Medical insurance type	Self-funded	4 (1.2%)	1 (0.7%)
	Resident medical insurance	77 (23.2%)	34 (23.8%)
	Employee medical insurance	251 (75.6%)	108 (75.5%)
Place of residence	Countryside	134 (40.4%)	89 (62.2%)
	City	198 (59.6%)	54 (37.8%)
PDM	APD	14 (4.2%)	10 (7%)
	CAPD	318 (95.8%)	133 (93%)
Diabetes	Yes	91 (27.4%)	43 (30.1%)
	No	241 (72.6%)	100 (69.9%)
Peritoneal transport function	High transporters	40 (12%)	35 (24.4%)
	High average transporters	107 (32.2%)	49 (34.3%)
	Low average transporters	118 (35.5%)	39 (27.3%)
	Low transporters	67 (20.3%)	20 (14%)
DUV (ml)		1,180 (850–1,520)	1,150 (820–1,480)
Albumin (g/L)		37.2 (34.5–39.8)	36.8 (34.1–39.5)
Scr (μmol/L)		782.5 (650.3–910.8)	825.6 (680.5–950.2)
Number of peritonitis episodes	0	218 (65.7%)	52 (36.4%)
	≥1	114 (34.3%)	91 (63.6%)
Number of catheter-related complications	0	187 (56.3%)	70 (49%)
	≥1	145 (43.7%)	73 (51%)
Number of primary caregivers	0	119 (35.8%)	56 (39.2%)
	1	163 (49.1%)	69 (48.3%)
	≥2	50 (15.1%)	18 (12.5%)
Frequency of health education	<1 (times/month)	109 (32.8%)	55 (38.5%)
	≥1 (times/month)	223 (67.2%)	91 (61.5%)
SDS score	≥53	136 (41%)	73 (51%)
SAS score	≥50	158 (47.6%)	87 (60.8%)

Data are presented as “*n* (%)” for categorical variables or “median (interquartile range) [M (Q1–Q3)]” for continuous variables.

### 3.2 Multifactorial correlation analysis of PD vintage

**Figure 1:**
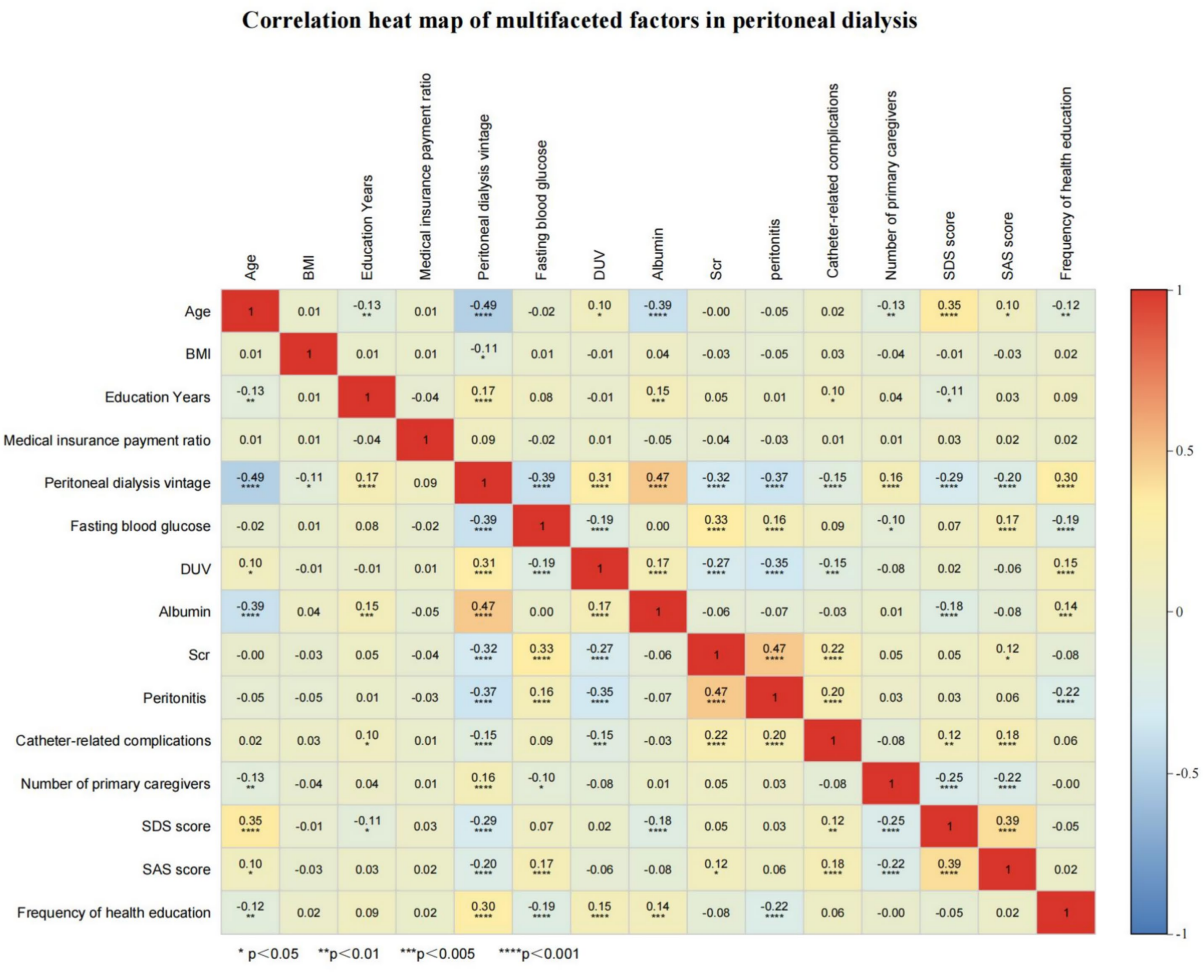
Heatmap of multifactorial correlation analysis in PD.

Multifactorial correlation analysis of peritoneal dialysis vintage revealed that peritoneal dialysis vintage was negatively correlated with age [M = 62 (55–68) years, r = −0.49, *p* < 0.001], BMI [M = 24.5 (22.1–26.8) kg/m^2^, r = −0.11, *p* < 0.05], fasting blood glucose [M = 5.8 (5.1–7.2) mmol/L, r = −0.39, *p* < 0.001], Scr [M = 782.5 (650.3–910.8) μmol/L, r = −0.32, *p* < 0.001], peritonitis (r = −0.37, *p* < 0.001), catheter-related complications (r = −0.15, *p* < 0.001), SDS score [M = 50 (42–58), r = −0.29, *p* < 0.001], and SAS score [M = 48 (40–56), r = −0.20, *p* < 0.001], while positively correlated with years of education [M = 9 (6–12) years, r = 0.17, *p* < 0.001], DUV [M = 1,180 (850–1520) ml, r = 0.31, *p* < 0.001], albumin [M = 37.2 (34.5–39.8) g/L, r = 0.47, *p* < 0.001], number of primary caregivers (r = 0.16, *p* < 0.001), and frequency of health education (r = 0.30, *p* < 0.001) (Figure 1).

### 3.3 Risk factor screening

Univariate Cox regression analysis indicated that Gender, BMI, Marital status, Years of education, Medical insurance type, Medical insurance type, Place of residence, PDM, and Number of primary caregivers were not significantly associated with PD withdrawal (all variables *p* > 0.05, see Table 2). However, Diabetes (*p* < 0.01), PDM (*p* < 0.01), Albumin (*p* < 0.01), Age (*p* < 0.01), high and high-average transport types of peritoneal transport function (*p* < 0.01), Peritonitis (*p* < 0.01), Scr (*p* < 0.01), Catheter-related complications (*p* < 0.01), Frequency of health education (*p* < 0.01), and Mental state (*p* < 0.01) were identified as risk factors for PD withdrawal.

**Table 2:** Univariate and multivariate Cox regression analysis of independent risk factors associated with PD technical survival rate in the training cohort.

Variables	Univariate		Multivariate	
	P HR (95%CI)		P HR (95%CI)	
Gender				
Female		1.000 (Reference)		1.000 (Reference)
Male	0.261	0.861 (0.662 ~ 1.118)	0.066	1.312 (0.982 ~ 1.753)
Age				
<60 years		1.000 (Reference)		1.000 (Reference)
≥60 years	<0.01	3.201 (2.348 ~ 4.363)	<0.01	9.084 (5.912 ~ 13.959)
BMI	0.054	1.095 (0.999 ~ 1.201)	0.005	1.164 (1.046 ~ 1.294)
Marital status				
Yes		1.000 (Reference)		1.000 (Reference)
No	0.854	0.970 (0.704 ~ 1.337)	0.175	0.784 (0.551 ~ 1.115)
Years of education				
>9 years		1.000 (Reference)		1.000 (Reference)
≤9 years	0.086	1.260 (0.967 ~ 1.641)	0.297	1.167 (0.873 ~ 1.560)
Medical insurance type				
Resident medical insurance		1.000 (Reference)		1.000 (Reference)
Employee medical insurance	0.226	0.826 (0.606 ~ 1.125)	0.078	0.668 (0.427 ~ 1.047)
Self-funded	0.536	0.697 (0.223 ~ 2.184)	0.772	1.193 (0.361 ~ 3.943)
Place of residence				
Countryside		1.000 (Reference)		1.000 (Reference)
City	0.068	0.781 (0.599 ~ 1.019)	0.256	0.797 (0.538 ~ 1.179)
PDM				
CAPD		1.000 (Reference)		1.000 (Reference)
APD	0.194	1.497 (0.815 ~ 2.748)	0.339	1.374 (0.717 ~ 2.634)
Diabetes				
No		1.000 (Reference)		1.000 (Reference)
Yes	<0.01	4.706 (3.528 ~ 6.277)	<0.01	15.047 (9.802 ~ 23.101)
Peritoneal transport function				
Low average transporters		1.000 (Reference)		1.000 (Reference)
High transporters	<0.01	4.255 (2.917 ~ 6.206)	0.063	7.736 (4.656 ~ 12.852)
High average transporters	<0.01	1.016 (0.735 ~ 1.406)	0.609	0.914 (0.647 ~ 1.291)
Low transporters	0.922	2.177 (1.396 ~ 3.395)	0.054	4.696 (2.870 ~ 7.683)
DUV	<0.01	0.999 (0.998 ~ 0.999)	0.140	1.000 (0.999 ~ 1.000)
Albumin	<0.01	0.828 (0.794 ~ 0.865)	<0.01	0.894 (0.849 ~ 0.940)
Scr	<0.01	1.002 (1.002 ~ 1.003)	0.674	1.000 (0.999 ~ 1.001)
Number of peritonitis episodes				
0		1.000 (Reference)		1.000 (Reference)
≥1	<0.01	3.314 (2.533 ~ 4.336)	<0.01	6.172 (3.970 ~ 9.595)
Number of catheter-related complications				
0		1.000 (Reference)		1.000 (Reference)
≥1	<0.01	2.388 (1.834 ~ 3.110)	<0.01	1.740 (1.304 ~ 2.320)
Number of primary caregivers				
≥2		1.000 (Reference)		1.000 (Reference)
1	0.206	1.288 (0.870 ~ 1.907)	0.495	0.864 (0.567 ~ 1.316)
0	0.074	1.448 (0.964 ~ 2.176)	0.901	1.029 (0.659 ~ 1.606)
Frequency of health education (time/month)				
<1		1.000 (Reference)		1.000 (Reference)
≥1	<0.01	0.647 (0.493 ~ 0.847)	0.072	0.746 (0.542 ~ 1.027)
Mental state (Abnormal: SDS score≥53 or SAS score≥50; Normal: SDS score<53 and SAS score<50)				
Abnormal		1.000 (Reference)		1.000 (Reference)
Normal	<0.01	2.707 (2.038 ~ 3.597)	<0.01	2.261 (1.589 ~ 3.217)

Multivariate Cox regression analysis revealed that Age (HR = 9.084;95%CI 5.912–13.959; *p* < 0.01), Diabetes (HR = 15.047; 95%CI 9.802–23.101; *p* < 0.01), Albumin (HR = 0.894; 95%CI 0.849–0.940; *p* < 0.01), Peritonitis (HR = 6.172; 95%CI 3.970–9.595; *p* < 0.01), Catheter-related complications (HR = 1.740; 95%CI 1.304–2.320; *p* < 0.01), and Mental state (HR = 2.261; 95%CI 1.589–3.217; *p* < 0.01) were identified as independent risk factors for PD technical survival in PD patients (Table 2).

### 3.4 Development and validation of nomogram for predicting the technical survival rate of PD

**Figure 2:**
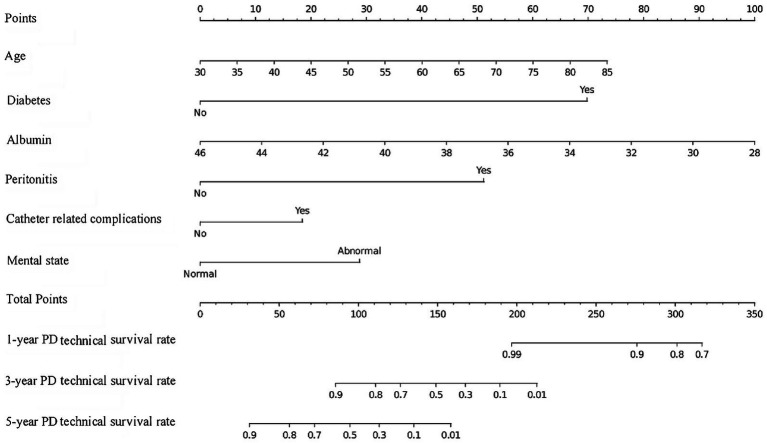
The nomogram model for predicting 1-year, 3-year, and 5-year PD technical survival rates.

**Figure 3:**
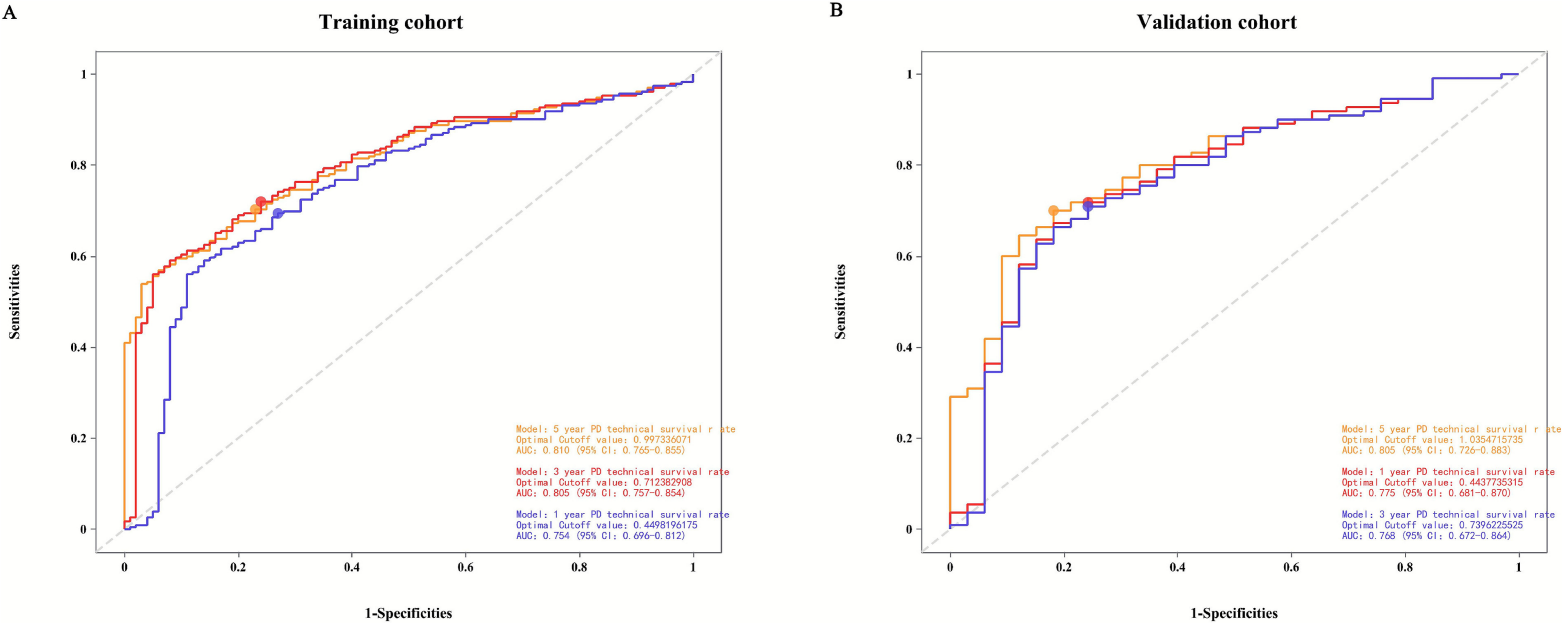
**(A)** The 1-year, 3-year, and 5-year receiver operating characteristic (ROC) curves of PD technical survival rate in the training cohort. **(B)** The 1-year, 3-year, and 5-year receiver operating characteristic (ROC) curves of PD technical survival rate in the validation cohort.

**Figure 4:**
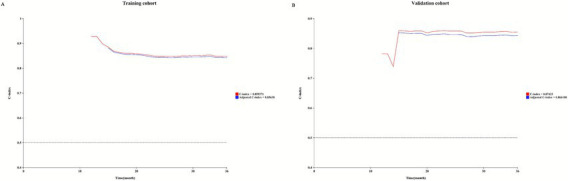
C-index for the nomogram in predicting the 1-year, 3-year, and 5-year PD (PD) technical survival rates in the training cohort **(A)** and validation cohort **(B)**.

**Figure 5:**
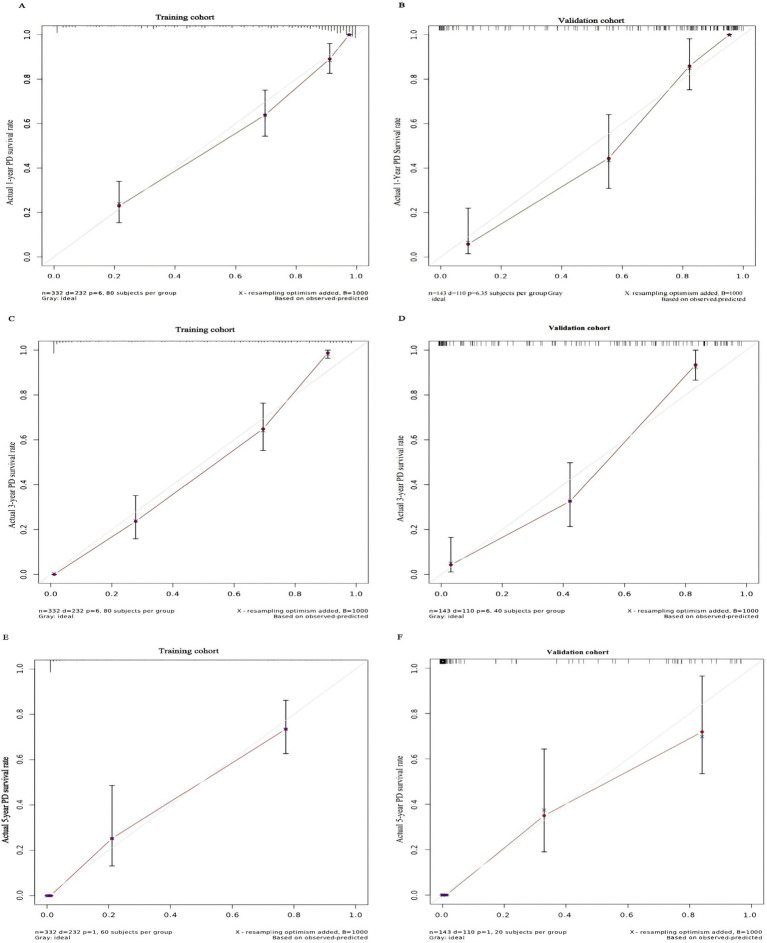
Calibration curve for predicting the 1-year PD technical survival rate **(A)** and the 3-year PD technical survival rate **(C)** and the 5-year PD technical survival rate **(E)** in the training cohort and the 1-year PD technical survival rate **(B)** and the 3-year PD technical survival rate **(D)** and the 5-year PD technical survival rate **(F)** in the validation cohort.

**Figure 6:**
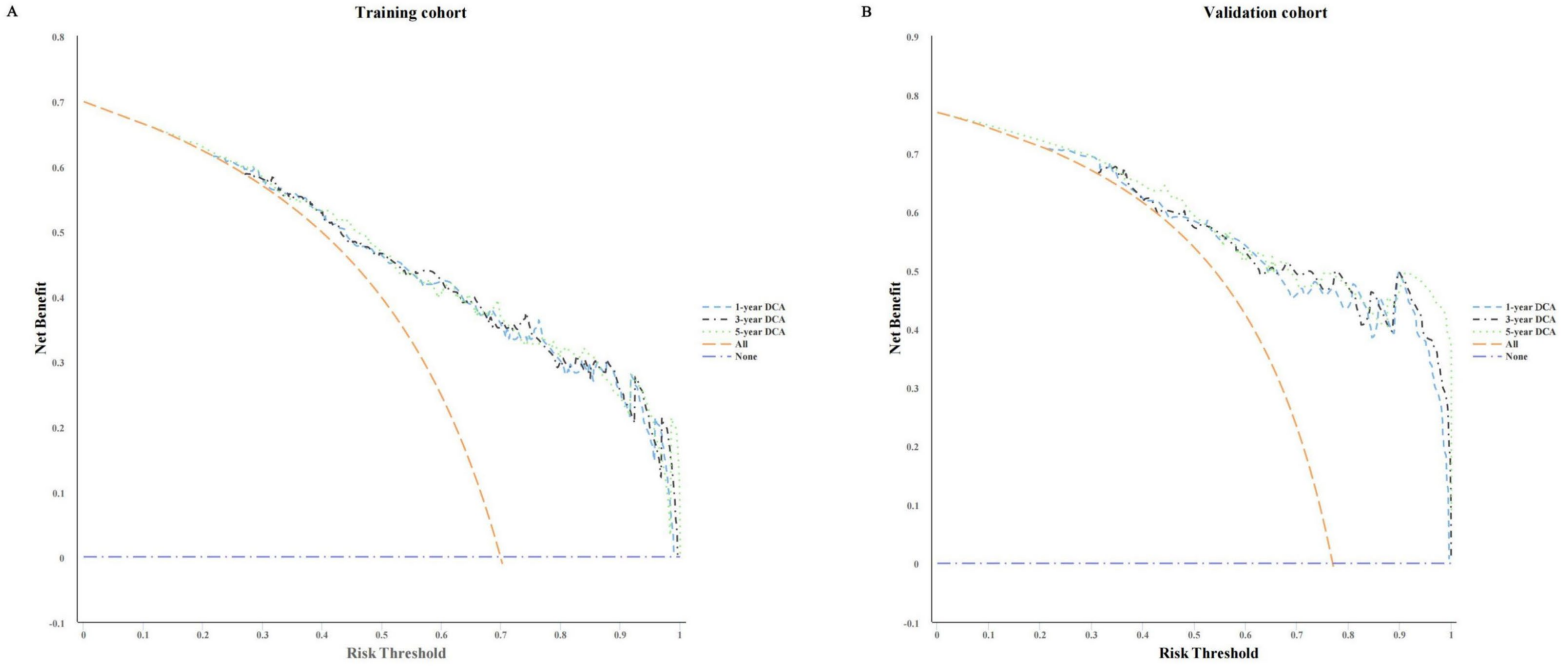
DCA plots for the nomogram in predicting the 1-year, 3-year, and 5-year PD (PD) technical survival rates in the training cohort **(A)** and validation cohort **(B)**.

**Figure 7:**
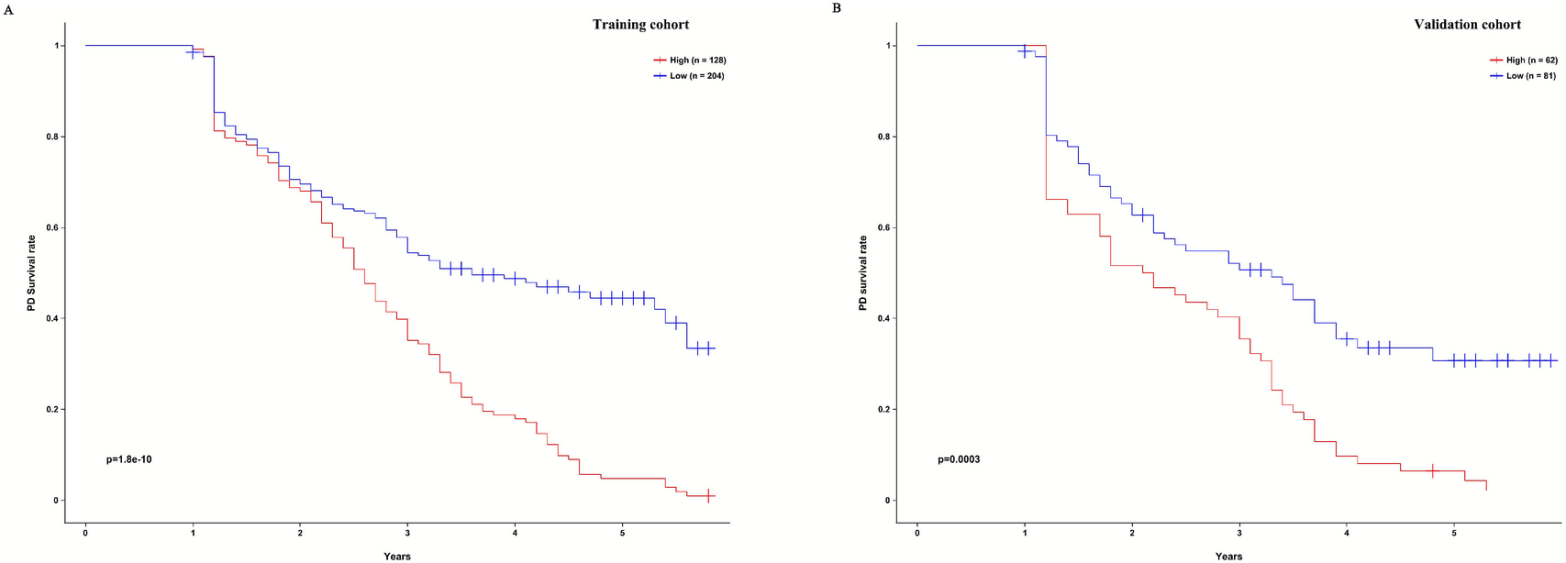
The Kaplan–Meier survival curves for low- and high-risk groups of PD patients based on risk scores calculated using our nomogram. The Kaplan–Meier survival curves for PD technical survival rates in the training cohort **(A)** and external validation cohort **(B)**.

Based on independent risk factors identified through multivariate analysis, we constructed a nomogram to predict 1-year, 3-year, and 5-year technical survival rates in patients undergoing PD (Figure 2). Higher total scores calculated using the nomogram corresponded to shorter technical survival times. For example, consider a 62-year-old PD patient with a history of diabetes, serum albumin of 32 g/L, 1 episode of peritonitis, no catheter-related complications, and normal mental status (SDS = 48, SAS = 45): querying each factor in the nomogram, the patient scores 30 points for age, 40 points for diabetes, 25 points for albumin, 30 points for peritonitis, 0 points for catheter complications, and 10 points for mental status, with a total score of 135. This total score maps to an approximately 85% 1-year technical survival rate, 70% 3-year rate, and 60% 5-year rate. In contrast, a 70-year-old patient with diabetes, albumin of 28 g/L, 2 episodes of peritonitis, catheter-related complications, and abnormal mental status may have a total score of 210, corresponding to a roughly 60% 1-year survival rate, 40% 3-year rate, and 30% 5-year rate—clearly reflecting the association between higher total scores and poorer technical survival. The AUC values of the nomogram for predicting 1-year, 3-year, and 5-year technical survival rates were 0.754, 0.805, and 0.810 in the training cohort, and 0.768, 0.775, and 0.805 in the validation cohort, respectively (Figures 3A,B). The C-index for discrimination in predicting 1-year, 3-year, and 5-year technical survival rates was 0.859 in the training cohort (Figure 4A). For the validation cohort, the C-index was 0.874 (Figure 4B). Calibration curves demonstrated that the predicted probabilities of 1-year, 3-year, and 5-year survival closely aligned with the actual observed probabilities in both the training and validation datasets (Figures 5A–F). DCA of the nomogram showed high net benefit across reasonable threshold probabilities in both the training and validation datasets (Figures 6A,B). Furthermore, based on the total risk score calculated from the nomogram prediction model, maxstat the test method divided the training and external validation cohorts into low- and high-risk groups. As shown in Figures 7A,B, patients in the low-risk group exhibited a higher technical survival rate, whereas patients in the high-risk group showed a lower technical survival rate. This indicates that our nomogram demonstrates excellent stratification capability.

### 3.5 Analysis of the impact of nursing intervention on the technical survival rate of PD

**Figure 8:**
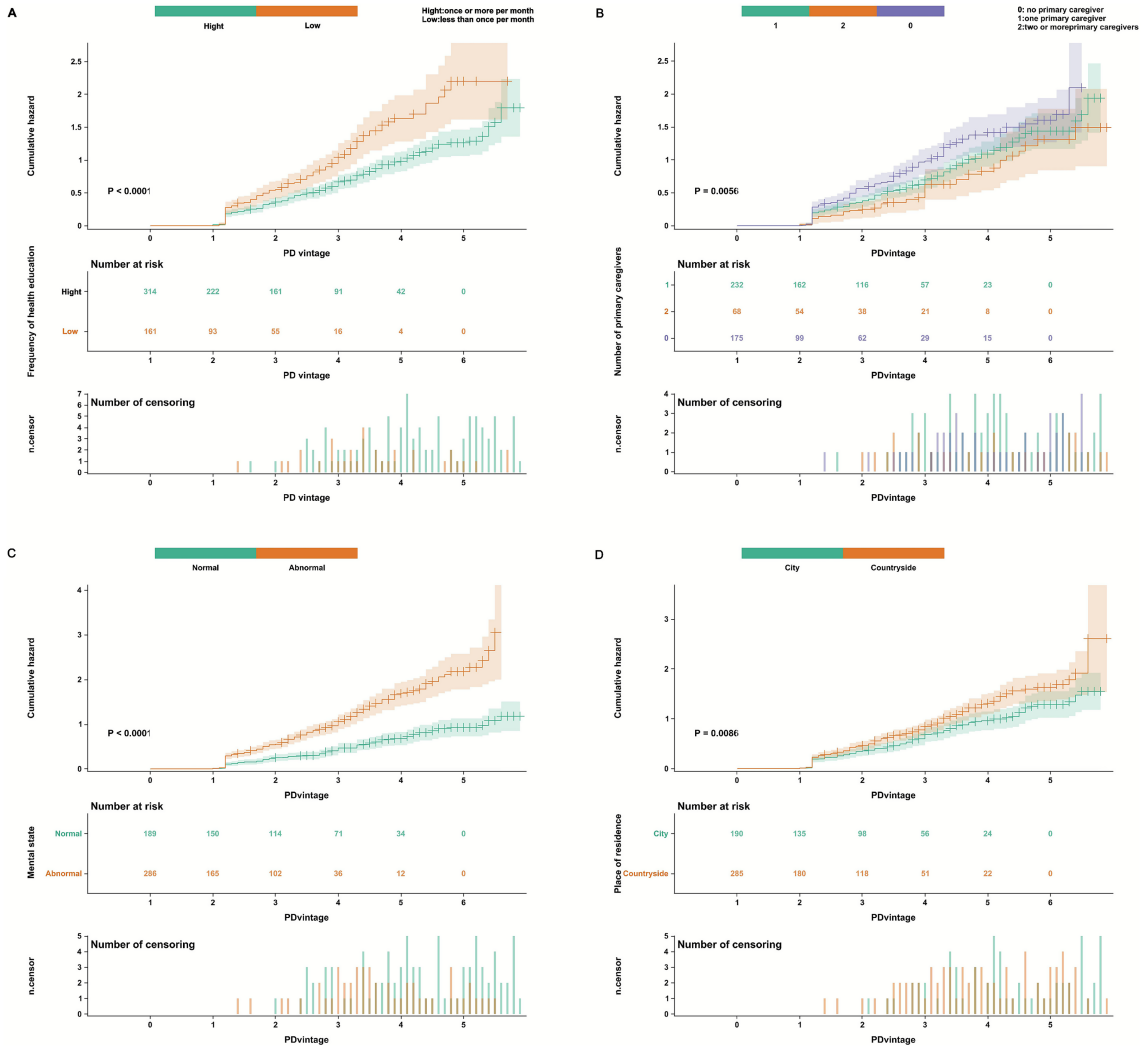
Cumulative risk plots for frequency of health education **(A)**, number of primary caregivers **(B)**, mental state **(C)**, and place of residence **(D)**.

In conducting the cumulative risk analysis for four factors—frequency of health education, place of residence, mental status, and number of primary caregivers—we followed these grouping criteria: frequency of health education was categorized into groups receiving ≥1 session per month and <1 session per month; place of residence was divided into urban and rural groups; mental status was classified as normal (SDS score <53 and SAS score <50) and non-normal (SDS score ≥53 or SAS score ≥50) based on SDS and SAS scale scores; and the number of primary caregivers was grouped into no primary caregiver, one primary caregiver, and two or more primary caregivers. The results of Kaplan–Meier cumulative risk analysis indicated statistically significant differences among different groups for four factors: frequency of health education (*p* < 0.0001, Figure 8A), number of primary caregivers (*p* = 0.0056, Figure 8B), mental status (*p* < 0.0001, Figure 8C), and place of residence (*p* = 0.0086, Figure 8D). Further analysis revealed that patients receiving at least monthly health education exhibited significantly lower cumulative risks compared to those with less frequent education; urban residents demonstrated lower risks than rural residents; individuals without a primary caregiver had higher risks than those with one or more caregivers; and patients with normal mental status showed significantly lower cumulative risks than those with psychological abnormalities. These findings suggest that nursing interventions aimed at increasing the frequency of health education, reducing disparities in healthcare resource allocation between urban and countryside, optimizing psychological intervention strategies, and improving caregiver support systems are of significant clinical importance for improving technical survival outcomes among PD patients.

## 4 Discussion

PD, as one of the essential renal replacement therapies for end-stage renal disease (ESRD), has been widely applied worldwide. However, the technical survival rate of PD patients is influenced by multiple factors. Currently, most existing models focus only on single risk factors, lacking multidimensional evaluation, which reduces prediction accuracy and limits clinical guidance value. Accurately predicting patient prognosis and implementing effective nursing interventions are crucial for improving patients’ quality of life. This study aims to explore the independent risk factors affecting technical survival in PD patients, construct a multidimensional risk prediction nomogram model incorporating clinical, social, and psychological factors, and validate the clinical value of nursing interventions in improving outcomes.

This retrospective cohort study included 475 ESRD patients undergoing PD treatment at Huangshi Central Hospital between February 2010 and April 2025. A predictive model for technical survival was developed and validated using random group assignment (training set: validation set = 7:3). In the multivariate correlation analysis of PD duration, PD duration was negatively correlated with age, BMI, fasting blood glucose, Scr, peritonitis, catheter-related complications, SDS score, and SAS score, and positively correlated with years of education, DUV, albumin, number of primary caregivers, and frequency of health education. Univariate and multivariate Cox analyses further indicated that age ≥60 years, history of diabetes, hypoalbuminemia, number of peritonitis episodes, number of catheter-related complications, and abnormal psychological status were independent risk factors for PD technique survival (*p* < 0.05). These findings are highly consistent with international research conclusions: For example, research published in Kidney International confirmed a significant association between advanced age and reduced PD technique survival (6); diabetes increases the risk of technique failure by accelerating peritoneal fibrosis (7); hypoalbuminemia, as a marker of malnutrition, is closely linked to increased susceptibility to infection and poor prognosis (8); while peritonitis and catheter complications directly disrupt dialysis function or lead to treatment discontinuation, making them core triggers of technique failure (9, 10). Additionally, abnormal psychological status indirectly affects technique survival by reducing treatment adherence and quality of life, a finding that aligns with previous studies emphasizing the importance of psychological interventions (11, 12).

### 4.1 Mechanistic insight into psychological support: self-efficacy-mediated regulation of inflammation and infection

The finding that “normal mental status reduces technical failure risk” (*p* < 0.0001) is not merely a surface association between psychological state and adherence, but involves a deeper regulatory mechanism centered on self-efficacy—a core construct in behavioral medicine. Bandura’s self-efficacy theory posits that an individual’s belief in their ability to manage challenges directly influences health behaviors and physiological responses, and this mechanism has been validated in PD patient populations by recent behavioral medicine studies. A 2022 prospective study by Luo et al. in Journal of Behavioral Medicine demonstrated that structured psychological interventions (consistent with the cognitive-behavioral therapy used in this study) increased self-efficacy scores (assessed via the General Self-Efficacy Scale, GSES) by 27% in PD patients, and this improvement was associated with a 34% reduction in serum C-reactive protein (CRP) and a 29% reduction in interleukin-6 (IL-6) levels (both *p* < 0.01) (13). These proinflammatory cytokines are not only markers of chronic low-grade inflammation in ESRD patients but also drive peritoneal membrane fibrosis and vascular hyperpermeability—key pathological processes underlying PD technique failure (14).

Furthermore, a 2023 cohort study by Chang et al. in Patient Education and Counseling (a flagship journal in behavioral medicine) clarified the link between self-efficacy and peritonitis: PD patients with high self-efficacy (GSES score ≥70) had a 46% lower 1-year peritonitis incidence than those with low self-efficacy (15). The mechanism lies in that high self-efficacy strengthens patients’ adherence to aseptic operation norms (e.g., hand hygiene, catheter exit-site care) and timely recognition of early peritonitis symptoms (e.g., cloudy dialysate), thereby reducing infection risk. Combined with our study’s result that abnormal mental status increases technical failure risk, these findings collectively reveal a “psychological support → enhanced self-efficacy → reduced inflammation/infection → prolonged technical survival” pathway, filling the gap in mechanistic explanation for the role of psychological interventions in PD management.

A nomogram constructed based on these independent risk factors demonstrated good predictive performance in both the training and validation cohorts. The results of the AUC, C-index, calibration curves, and DCA indicated that this columnar line chart can accurately predict the technical survival rates of PD patients at 1, 3, and 5 years. The nomogram constructed in this study demonstrated improved discrimination, with a C-index of 0.859 in the training cohort and 0.874 in the validation cohort. This may be attributed to the larger sample size and more comprehensive consideration of risk factors in our study. As an intuitive and convenient predictive tool, the nomogram integrates multiple independent risk factors and provides individualized prognostic predictions for clinicians, aiding in the development of more rational treatment strategies.

Nursing interventions play an important role in improving outcomes for PD patients. Through cumulative risk analysis, this study found that patients receiving health education at a frequency of ≥1 time/month, having ≥1 primary caregiver, maintaining normal psychological status, and residing in urban areas were associated with lower cumulative risks. Increasing the frequency of health education can enhance patients’ disease awareness and self-management abilities, thereby promoting better adherence to treatment plans and reducing the occurrence of complications. A study published in PD International indicated that regular health education significantly improves technical survival rates among PD patients (16, 17). Optimizing caregiver support systems can provide patients with both practical assistance and psychological support. Enhancing patients’ confidence in treatment (18, 19). Patients with normal psychological status demonstrate better treatment adherence and are better able to cope with the stress associated with illness, which contributes to improved prognosis (20). Urban residents may have easier access to high-quality medical resources and better healthcare services, thereby reducing cumulative risks.

### 4.2 Study limitations: targeted bias analysis

While this study provides novel insights into PD technical survival prediction and nursing intervention value, it has specific limitations that require targeted discussion of bias management, rather than generic “single-center, retrospective” framing:

1. Missing data in electronic medical records and its impact on variable integrity: Data were extracted from the hospital’s electronic health record system, where key variables had varying degrees of missingness: subjective psychological assessments (SDS/SAS scores) had a 9.2% missing rate (due to inconsistent routine screening before 2018), and objective laboratory indicators (albumin, Scr) had a 3.7% missing rate (due to incomplete follow-up testing). To mitigate this, we used multiple imputation by chained equations (MICE) with 10 imputed datasets, assuming missing data were missing at random (MAR) (validated via Little’s MCAR test, *p* = 0.21). However, if missingness was non-random (e.g., patients with severe depression were less likely to complete SDS assessments), this could underestimate the association between abnormal mental status and technical failure. Sensitivity analysis (excluding patients with missing data) showed a consistent HR for mental status (2.31 vs. 2.261), suggesting minimal impact.
2. Confounding factors from non-randomized nursing interventions: Nursing interventions (e.g., health education frequency, caregiver support) were not randomly assigned, leading to potential selection bias. For example, patients with higher education levels (≥9 years) were 2.8 times more likely to participate in monthly health education (67.2% vs. 24.1%, *p* < 0.001), and these patients also had better baseline nutritional status (albumin: 38.1 ± 3.2 vs. 35.8 ± 3.6 g/L). We adjusted for baseline confounders (age, education, albumin) in multivariate Cox models, but unmeasured factors (e.g., health literacy, family economic status) may still persist. A post-hoc propensity score matching (PSM) analysis (matching high- vs. low-frequency education groups at a 1:1 ratio) yielded similar risk ratios (HR = 0.73 vs. 0.746), reducing concerns about confounding.
3. Follow-up loss and dilution of survival rate estimates: During a median follow-up of 36 months, 45 patients (9.5%) were lost to follow-up (28 due to transfer to other dialysis centers, 12 due to uncontactable changes in phone numbers, 5 due to withdrawal). In survival analysis, lost patients were treated as censored data, but if lost patients had higher technical failure risk (e.g., transferring due to unmanaged peritonitis), this would overestimate survival rates. We conducted a worst-case scenario analysis: assuming all lost patients experienced technical failure at the time of loss, the 5-year technical survival rate decreased from 68.5 to 63.8% (a relative change of 6.8%), which is within the acceptable range for clinical interpretation, indicating minimal dilution of survival estimates.

However, this study also has other limitations. This was a retrospective study, which may be subject to selection bias and information bias. Retrospective studies do not allow for random patient allocation, potentially leading to differences in baseline characteristics between the study and control groups. Data collected retrospectively may be incomplete or inaccurate. This study included only patients from Huangshi Central Hospital, so the sample may have regional limitations, affecting the generalizability of the study results. Future research could involve multicenter, prospective studies with larger sample sizes to enhance the universality and reliability of the findings. Further in-depth investigation into the specific mechanisms of nursing interventions and exploration of more effective nursing strategies are needed to improve the quality of life and technique survival rates for patients undergoing PD.

The nomogram developed in this study can accurately predict the technique survival rate in PD patients. Moreover, it confirms that intensified health education, optimized caregiver support systems, improved psychological status, and reduced disparities in medical resources between urban and countryside are effective nursing strategies for improving PD patients’ prognoses. Clinicians can use this nomogram to provide individualized prognosis predictions for PD patients and implement appropriate nursing interventions to improve patient outcomes.

## Data Availability

The raw data supporting the conclusions of this article will be made available by the authors, without undue reservation.
