# Supplementary material for: Analysis of prognostic prediction and nursing intervention value in PD patients based on nomogram model
Source: Front Med (Lausanne). 2025 Dec 15;12:1645631. doi: 10.3389/fmed.2025.1645631 (PMC12745274; doi:10.3389/fmed.2025.1645631)
Supplement: Supplementary file 1 [file Table_1.DOCX]

Supplementary Material

# **Details of Standardized Nursing Interventions for Peritoneal Dialysis (PD) Patients**

# This supplementary material provides comprehensive information on the standardized nursing interventions implemented in the study, including health education, psychological support, and primary caregiver training. All interventions were developed in accordance with the 2022 International Society for Peritoneal Dialysis (ISPD) Guidelines for PD Patient Care and Education[1], and designed to address key risk factors identified in the study (e.g., abnormal mental state, insufficient caregiver support, low health literacy).

## 1. Health Education Intervention

**1.1 Target Population**

### All enrolled PD patients (regardless of baseline characteristics) to improve disease awareness, self-management ability, and adherence to treatment.

**1.2 Key Components (Core Content)**

Aligned with the study’s focus on reducing complications (peritonitis, catheter-related issues) and optimizing treatment adherence, the education content included:

1.PD Technical Operations: Step-by-step guidance on aseptic fluid exchange (e.g., hand hygiene, connection/disconnection of the PD catheter, disposal of used dialysate), catheter care (daily cleaning, dressing change, prevention of kinking/obstruction), and recognition of abnormal dialysate (e.g., cloudy, bloody, or foul-smelling effluent).

2.Complication Prevention: Early identification of peritonitis (fever, abdominal pain, cloudy effluent) and catheter-related infections (redness, swelling, drainage at the catheter exit site), plus immediate action steps (e.g., contacting the PD team, collecting effluent samples for testing).

3.Diet and Medication Management: Individualized dietary guidance (low-salt, low-phosphorus, low-potassium diets; protein intake adjustment based on albumin levels) and medication adherence education (e.g., antihypertensives, phosphate binders, and erythropoietin—importance of regular intake and avoiding missed doses).

4.Self-Monitoring: Training on daily recording of body weight, blood pressure, urine output, and dialysate ultrafiltration volume; recognition of fluid overload (edema, shortness of breath) and strategies for adjustment.

**1.3 Implementation Methods**

Format: Combined one-on-one personalized guidance (for technical operations) and monthly group lectures (for general knowledge, e.g., diet/medication); supplemented with video tutorials (accessible via hospital WeChat platform) and illustrated manuals (simplified for low-education or rural patients, addressing urban-rural disparities noted in the study).

Frequency: ≥1 session/month (consistent with the study’s finding that this frequency reduces cumulative risk; P<0.0001); additional ad hoc sessions provided for patients with poor knowledge retention (e.g., failed post-education quizzes).

Duration: 30–45 minutes per session (one-on-one) or 60 minutes per group lecture.

**1.4 Quality Control**

Post-education quizzes (80% pass rate required) to assess knowledge mastery; retraining provided for those who fail.

Monthly review of patients’ self-monitoring records to verify compliance with technical operations and diet/medication plans.

**2. Psychological Support Intervention**

**2.1 Target Population**

PD patients with abnormal mental state (defined as Self-Rating Depression Scale [SDS] score ≥53 or Self-Rating Anxiety Scale [SAS] score ≥50), as this group was identified as an independent risk factor for poor technical survival (HR=2.261, 95%CI:1.589–3.217; P<0.01).

2.2 Key Components (Core Content)

Based on cognitive-behavioral therapy (CBT), adapted for PD patients:

Emotion Identification and Regulation: Helping patients recognize symptoms of depression/anxiety (e.g., persistent low mood, sleep disturbances, irritability) and teaching relaxation techniques (deep breathing, progressive muscle relaxation) to alleviate acute stress.

Cognitive Restructuring: Correcting negative cognitions about PD (e.g., “PD limits my life” → “PD allows me to maintain daily activities with proper management”) and building confidence in long-term treatment.

Social and Family Linkage: Collaborating with patients’ families to create a supportive home environment (e.g., encouraging participation in daily activities, avoiding excessive protection) and connecting patients with PD support groups (in-person or online) to reduce loneliness.

**2.3 Implementation Methods**

Format: One-on-one psychological counseling (conducted by a certified clinical psychologist or trained psychiatric nurse); referrals to the hospital’s psychiatry department for patients with severe symptoms (e.g., SDS ≥63 or suicidal ideation) for further evaluation and medication adjustment (if needed).

Frequency: Initial phase (Weeks 1–4): 1 session/week; maintenance phase: 1 session every 2 weeks until SDS <53 and SAS <50 (sustained for 2 consecutive months).

Duration: 40–50 minutes per counseling session.

**2.4 Quality Control**

Monthly reassessment of SDS/SAS scores to monitor symptom improvement; adjustment of counseling frequency based on progress.

Documentation of counseling content and patient feedback to ensure consistency in intervention delivery.

**3. Primary Caregiver Support Training**

**3.1 Target Population**

Primary caregivers of PD patients (≥1 caregiver per patient, as the study found that patients with no caregivers had higher cumulative risk; P=0.0056). Caregivers included family members (spouses, children) or professional caregivers.

**3.2 Key Components (Core Content)**

Focused on equipping caregivers to assist with PD management and support patient well-being:

PD Auxiliary Operations: Training on assisting patients with fluid exchange (for those with limited mobility), monitoring dialysate volume and physical characteristics (e.g., reporting cloudy effluent to the PD team), and maintaining the patient’s PD supplies (e.g., storing dialysate at room temperature, checking expiration dates).

Emergency Response: Step-by-step guidance for handling acute events (e.g., catheter dislodgment: applying pressure to the exit site, contacting emergency services; peritonitis symptoms: helping the patient measure temperature and collect effluent samples).

Patient Support Collaboration: Teaching caregivers to recognize changes in the patient’s mental state (e.g., withdrawal, irritability) and collaborate with the psychological support team; assisting with dietary adherence (e.g., preparing low-salt meals) and medication reminders.

**3.3 Implementation Methods**

Format: One-on-one hands-on training (conducted by a senior PD nurse) at the patient’s bedside (during hospital admission or outpatient follow-up); supplemented with a caregiver manual and video demonstrations of emergency procedures.

Frequency: Initial training (1 session) before the patient starts home PD; refresher training (1 session) every 3 months to reinforce skills and address new questions.

Duration: 45–60 minutes per training session (including 20 minutes of hands-on practice).

**3.4 Quality Control**

Practical skill assessments (e.g., demonstrating catheter dressing change, describing emergency steps for peritonitis) to ensure caregivers can independently assist the patient; retraining provided for those who fail.

Quarterly caregiver satisfaction surveys to identify gaps in training content (e.g., additional support for nighttime emergency management).

**4. Intervention Delivery Team and Supervision**

Team Composition: Senior PD nurses (with ≥5 years of PD care experience) for health education and caregiver training; clinical psychologists (with CBT certification) for psychological support. All team members completed 8 hours of training on the 2022 ISPD Guidelines before intervention implementation.

Supervision: A monthly meeting of the intervention team to review case records, discuss challenges (e.g., low adherence in rural patients), and adjust intervention strategies (e.g., adding dialect-specific materials for rural caregivers).

**Reference**

[1] Piraino B, Bailie GR, Bernardini J, et al. Peritoneal dialysis-related infections recommendations: 2022 update[J]. Perit Dial Int, 2022, 42(3): 207-247.
